# The Role of Additive Neurogenesis and Synaptic Plasticity in a Hippocampal Memory Model with Grid-Cell Like Input

**DOI:** 10.1371/journal.pcbi.1001063

**Published:** 2011-01-27

**Authors:** Peter A. Appleby, Gerd Kempermann, Laurenz Wiskott

**Affiliations:** 1 School of Computing, University of Leeds, Leeds, United Kingdom; 2 CRTD - Center for Regenerative Therapies Dresden, Dresden, Germany; 3 DZNE, German Center for Neurodegenerative Disease, Dresden, Germany; 4 Institut für Neuroinformatik, Ruhr-Universität Bochum, Bochum, Germany; University of Freiburg, Germany

## Abstract

Recently, we presented a study of adult neurogenesis in a simplified hippocampal memory model. The network was required to encode and decode memory patterns despite changing input statistics. We showed that additive neurogenesis was a more effective adaptation strategy compared to neuronal turnover and conventional synaptic plasticity as it allowed the network to respond to changes in the input statistics while preserving representations of earlier environments. Here we extend our model to include realistic, spatially driven input firing patterns in the form of grid cells in the entorhinal cortex. We compare network performance across a sequence of spatial environments using three distinct adaptation strategies: conventional synaptic plasticity, where the network is of fixed size but the connectivity is plastic; neuronal turnover, where the network is of fixed size but units in the network may die and be replaced; and additive neurogenesis, where the network starts out with fewer initial units but grows over time. We confirm that additive neurogenesis is a superior adaptation strategy when using realistic, spatially structured input patterns. We then show that a more biologically plausible neurogenesis rule that incorporates cell death and enhanced plasticity of new granule cells has an overall performance significantly better than any one of the three individual strategies operating alone. This adaptation rule can be tailored to maximise performance of the network when operating as either a short- or long-term memory store. We also examine the time course of adult neurogenesis over the lifetime of an animal raised under different hypothetical rearing conditions. These growth profiles have several distinct features that form a theoretical prediction that could be tested experimentally. Finally, we show that place cells can emerge and refine in a realistic manner in our model as a direct result of the sparsification performed by the dentate gyrus layer.

## Author Summary

Contrary to the long-standing belief that no new neurons are added to the adult brain, it is now known that new neurons are born in a number of different brain regions and animals. One such region is the hippocampus, an area that plays an important role in learning and memory. In this paper we explore the effect of adding new neurons in a computational model of rat hippocampal function. Our hypothesis is that adding new neurons helps in forming new memories without disrupting memories that have already been stored. We find that adding new units is indeed superior to either changing connectivity or allowing neuronal turnover (where old units die and are replaced). We then show that a more biologically plausible mechanism that combines all three of these processes produces the best performance. Our work provides a strong theoretical argument as to why new neurons are born in the adult hippocampus: the new units allow the network to adapt in a way that is not possible by rearranging existing connectivity using conventional plasticity or neuronal turnover.

## Introduction

The adult mammalian brain contains two neurogenic regions, the hippocampus and the olfactory bulb. One important distinction between these two areas is that neurogenesis in the olfactory bulb is thought to be part of a turnover of cells while neurogenesis in the dentate gyrus is believed to be an additive process where new units are added to an expanding network [1]–[4]. Thousands of new granule cells are produced each day in the dentate gyrus of young adult animals, a number that declines sharply as the animal ages [5]–[9]. Although the majority of the new neurons die off a subset is incorporated into the dentate gyrus and become fully functional units incorporated into the existing network [10]–[12]. Surviving granule cells have been shown to persist for at least a year [2]. In the course of their development the new granule cells go through a period of enhanced synaptic plasticity [13]–[16] and a critical time window for their recruitment for long-term survival [17], [18] as well as their relevance for performance in selected behavioural tasks [19].

Computational models have made great progress in understanding the functional relevance of adult-born neurons. Models of hippocampal networks that include adult neurogenesis have examined neurogenesis as either part of a neuronal turnover [20]–[26] or, more recently, as part of an additive process [27], [28]. These studies show that incorporating neurogenesis into a network can be advantageous in number of learning tasks, for example when a network is required to learn a new set of input-output relationships that overwrite a previously learned set of relationships, or when a network must learn to distinguish very similar inputs patterns (an ability known as pattern-separation). In our own work we have examined the functional role of additive neurogenesis in the rat dentate gyrus by modeling neurogenesis in a simplified memory model of the hippocampus [29], [30]. The network incorporated both a divergence in unit number between the EC and DG and sparse coding in the DG, both notable features of the hippocampus. We required the system to correctly encode and decode memory patterns under the constraint that the input statistics change over time. Such a change in input statistics might occur due to a change in the external environment, for example when an animal moves from one laboratory enclosure to another, producing different firing correlations in the entorhinal cortex. In order to deal with these new correlations and correctly encode patterns drawn from the new input statistics the network typically must adapt its internal connectivity. We found that introducing a conventional form of plasticity, where existing synaptic connections can change over time, accommodated the new input statistics from the novel environment but led to a breakdown of network function when retrieving and decoding previously laid down memory patterns, a problem we referred to as catastrophic interference between old and new encodings. Adaptation strategies based on neuronal turnover, where neurons in the network are allowed to die and be replaced, suffered from similar problems for essentially the same reasons. An adaptation strategy based on additive neurogenesis, on the other hand, allowed the network to accommodate the new input statistics while at the same time preserving representations of earlier environments. This came at the expense of a lower representational power in the original environment due to the network having initially fewer intermediate layer units, but the increase in retrieval accuracy far outweighed this loss so that the network as a whole operated with a much higher fidelity. Thus, additive neurogenesis allowed the network to adapt to changes in input statistics while preserving the retrieval properties of the network and eliminating entirely the problem of interference. We concluded that there are strong theoretical arguments as to why additive neurogenesis should be observed in the dentate gyrus of the hippocampus as it endows the network with the ability to adapt in a way that is not possible with conventional plasticity or neuronal turnover.

A notable feature of this earlier model was the use of multidimensional Gaussian input distributions to model the input firing patterns that arrived at the entorhinal cortex input layer. We made this choice for reasons of simplicity and analytical tractability. This choice did, however, create a degree of arbitrariness in both the definition of input statistics for a single environment and also in how those statistics changed between environments. This arbitrariness meant it was difficult to directly link our results to experimental data on, for example, the amount of neurogenesis observed experimentally in real animals (in our case, rats). The lack of any spatial structure in our input and any analogue of spatial position also meant that neurons in the DG that respond to a specific spatial location of the animal (known as “place cells”) were completely absent from our model.

Here, we extend our earlier model to incorporate realistic, spatially driven input firing patterns in the EC in the form of grid cells. Grid cells have been well documented in the dorsocaudal region of the medial entorhinal cortex of awake and behaving rats, and are comparatively well characterised experimentally [31]–[33]. Importantly for our model, the manner in which the grids change when the rat enters a new environment has also been examined. Thus, we may draw directly upon experimental data to build a phenomenological model of grid cell firing and to define the manner in which those statistics change when the animal enters a new environment. This allows us to generate realistic input statistics and to evaluate network performance in a computational task that is much more closely related to that of the real hippocampus. This allows us to connect our results, including the time course of neurogenesis over the lifetime of an animal, more closely to experimental data. It also provides us with a direct analogue of spatial position in our model which allows us to explore spatial properties of the network such as the appearance and refinement of place cells in a way that was not previously possible.

## Model

**Figure 1:**
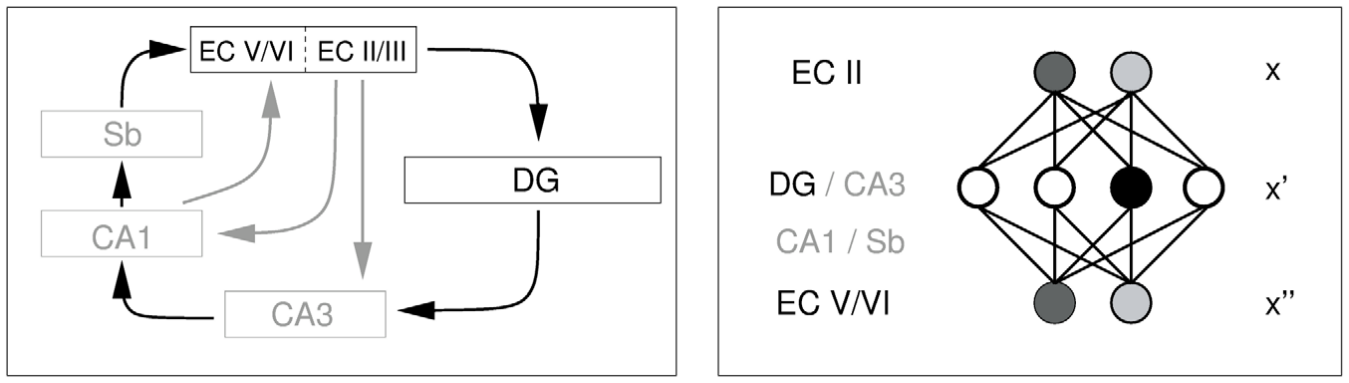
Our simplified hippocampal model. Left panel: We focus on the role of the EC and DG, while the remaining areas are modeled only implicitly and are shown as grey in the figure. Connectivity that does not play a role in our model is indicated by grey arrows. Right panel: the autoencoding network we abstract from our simplified model. A continuous 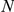-dimensional EC input pattern, 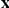, is generated from a phenomenological model of grid cell firing and encoded into a binary 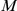-dimensional DG representation, 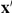. The encoded pattern is stored and later retrieved, then inverted to reproduce a continuous approximation to the original pattern, 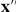. The networks we simulate in the results section have 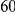 units in the input layer and up to 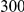 units in the hidden layer.

We are interested in examining the functional consequences of additive neurogenesis in as wide a sense as possible. We therefore consider a generalised memory model that is compatible with a number of existing models of hippocampal function. This simplified hippocampal memory model is described in detail in Appleby and Wiskott (2009) and is illustrated in Figure 1. Briefly, we make the hypothesis that the hippocampus acts as a temporary memory system. We focus on the role of the EC and DG layers in this system. We do not include areas downstream of the DG, such as CA3 and CA1, which are left implicit in our model. Incoming patterns arrive in layers II and III of the EC and are then encoded by the DG. Units in the EC have a graded response, so their activity is a real-valued number. Units in the DG are binary, so their activity can be zero or one, a choice that reflects the bursting nature of cells in the DG. Activity in the DG is governed by a winner-takes-all algorithm, so that only one unit in the DG is active at any one time, which reflects the extremely sparse activity levels in the DG [34], [35]. Each unit 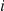 in the DG layer has an associated 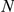-dimensional *encoding vector* 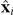 and encoding of EC patterns takes place using a simple winner-takes-all mechanism. Each input vector activates the unit in the hidden layer that has an encoding vector lying closest to it. In other words, the activation of the DG unit 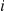 is given by
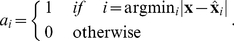
(1)This activation rule induces a Voronoi tessellation of the input space into 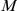 Voronoi cells [30]. Encoded patterns are stored downstream, possibly in area CA3, and later retrieved and decoded via an associated *decoding vector* 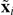, which determines the output vector 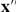. Typically, en- and decoding vectors are identical, so that 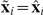, but if the network adapts at any time between storage and retrieval the decoding vectors used during retrieval might be different from the encoding vectors used during storage.

We measure the performance of the network with the mean squared Euclidean distance between input vectors 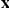 and output vectors 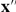,
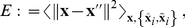
(2)where the averaging denoted by 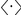 is over the distribution of the input vector 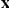, the sets of encoding vectors 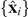 with 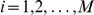, and the sets of decoding vectors, 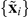, if they differ from the encoding vectors.

We consider a scenario where a virtual rat moves through a sequence of 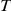 environments labeled 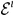, where 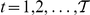. When fully adapted to environment 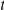 the network is referred to as *network* 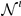. After the network has subsequently adapted to environment 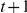 it is referred to as *network* 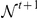. As the rat moves through this sequence of environments we quantify the performance of the network using two kinds of error.

- **Recoding error**, which is the error for patterns stored and retrieved with network 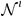 in environment 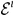.
- **Retrieval error**, which is the error when patterns stored with network 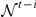 in environment 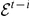, 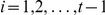, are later retrieved and decoded with network 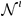.

It is important to distinguish these two errors as the internal state of the network will typically not be the same in environment 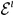 as it was in environment 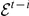 and the retrieval error will therefore not necessarily be the same as the recoding error when the pattern was initially stored.

### Spatial input and grid cells

The activity of granule cells in the DG is known to have a strong spatial dependence. Typically cells respond very strongly at a small number of specific spatial locations in the spatial environment, referred to as place fields, but are quiescent otherwise [36]. Experimental work has shown that there is also a strong spatial dependence in the activity of layer II of the dorsocaudal medial EC, an area upstream of the DG which provides much of its input [31]–[33]. Cells in this region of the EC display very regular topographically organised firing patterns that map the spatial environment. This topographic map is in the form of a regular triangular lattice that covers the entire spatial environment. In contrast to place cells, which tend to have a single or very few firing locations in any one environment, cells in the EC display highly elevated (although not necessarily identical) firing rates at any vertex in the triangular lattice, which has inspired the name “grid cells”.

**Figure 2:**
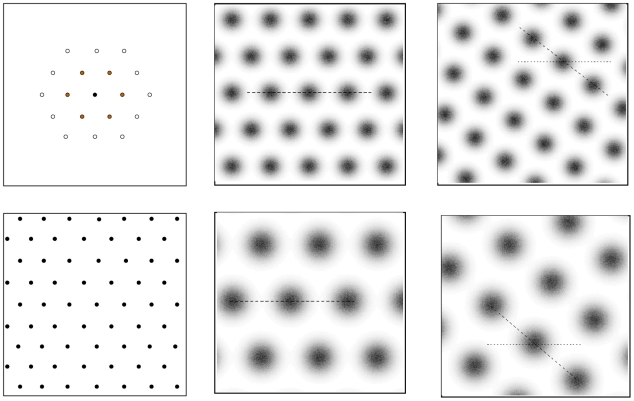
Topographic firing patterns in the EC. Top left panel: Formation of a grid governing the firing of a particular EC cell. A single vertex is placed at the specified grid origin (solid circle) which we choose for this example to be at the centre of the environment, then surrounded by six further vertices at the specified grid spacing (grey-filled circles). These vertices in turn are surround by twelve further vertices (white-filled circles) which begins to cover the spatial environment with a grid of equilateral triangles. Bottom left panel: Completed grid covering the entire spatial environment. In our simulations, the grid is extended to 1 m beyond the boundary wall to minimise edge effects. Middle panels: Two example grids in environment 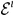. Firing rates range from zero Hertz (white) to twelve Hertz (black). The dashed lines indicate the “centre line” of each grid which passes through the grid origin. The grids have different origins as well as vertex spacings and field sizes, but similar orientations. Right panels: The same two grids after entry to environment 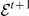. The grids have undergone a coherent rotation of grid orientation and independent random shifts in grid origin. The dashed lines show the new grid centre lines in environment 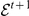 superimposed on the (unrotated) centre line from the previous environment 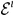, shown as a dotted line.

To introduce spatially driven activity in our model we define a set of triangular grids that will determine firing patterns in the EC as the rat explores its environment. To do this we require an origin and orientation for the grid, a vertex spacing, and a description of the peak firing rate and field sizes at each vertex. We may then construct a triangular lattice by placing a central vertex at the specified origin and placing six further vertices around it at the specified vertex spacing and orientation. This process is then repeated until the whole of the spatial environment is covered, as illustrated in the left two panels of Figure 2. Repeating this process for each of the 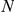 cells in the input layer gives us a set of 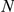 grids that together determine activity in the EC in a particular environment as a function of spatial location.

As the topographic maps that govern grid cell firing have been experimentally well characterised we may draw directly upon experimental data to build our model [31]–[33]. The main advantage of this approach is that it reduces the degree of arbitrariness in our choice of input patterns and allows us to explore network performance in a much more realistic encoding-decoding task. Grid spacing and field radii are topographically ordered throughout the EC with strong correlations between neighbouring cells. Grid spacing increases linearly from around 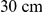 to 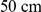 when recording from the dorsal to ventral ends of the EC [32]. Field radius ranges from 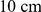 to 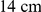 and increases with the grid spacing along the same dorsoventral axis. In contrast, the orientations and peak locations of the grids are apparently unstructured and are drawn from the whole range of values along the dorsoventral axis, although anatomically adjacent cells do appear to share very similar orientations. Peak firing rates are also apparently unstructured but the mean firing rate of the overall population follows a Gamma-like distribution [31].

Although individual cells in layer II of the EC arborise over a considerable fraction of the DG, anatomically adjacent cells are much more likely to share the same innervation targets. For reasons of computational tractability we simulate a group of neighbouring EC cells which permits us to use all-to-all connectivity, so that every unit in the input layer is connected to every unit in the hidden layer. We assume that this ensemble of EC input cells comes from the mid region of the dorsocaudal medial EC and take mid-ranged values as appropriate. We simulate a 1 m by 1 m environment for our virtual animal to explore, an enclosure size similar to that used in the experimental literature from which we draw our parameters.

In summary, the parameters describing the grids are generated according to the following scheme.

- **Grid origin:** Independently drawn from a uniform distribution bounded by zero and one.
- **Grid spacing:** Independently drawn from a Gaussian distribution with mean 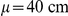 and standard deviation 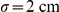.
- **Grid orientation:** One global value drawn from a uniform distribution bounded by zero and 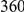. Each grid is then subjected to independent Gaussian noise with mean 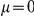 degrees and standard deviation 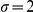 degrees.
- **Field size:** Independently drawn from a Gaussian distribution with mean 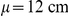 and standard deviation 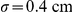

- **Peak firing rate:** One global value drawn from a Gamma distribution with shape parameter 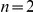 and scale parameter 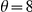. Each individual peak is subject to Gaussian noise with mean 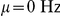 and standard deviation 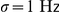.

The resulting vertices form a regular lattice of equilateral triangles which extends across the entire spatial environment. Representative examples are illustrated in the middle two panels of Figure 2. Firing rates for the EC cells are generated by summing the contributions from all the grid vertices spanning the entire extended environment. Although we limit our spatial environment to a 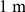 by 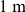 box we extend the grids an additional 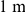 beyond the box boundary to minimise edge effects produced by vertices moving into and out of the environment.

#### Grid cell remapping

Our model of grid-cell firing allows us to use realistic input statistics when generating EC input patterns for the DG to encode. In addition to modeling the grid cells themselves we are interested in how the grids change when the rat moves into a new environment. Changes in the external environment are known to drive distinct changes in activity patterns in hippocampal areas downstream of the EC, an example being rate- or global-remapping of place cells. Rate-remapping is triggered by a limited change in the environment such as changing the colour of the walls of the enclosure [33]. Global-remapping is triggered by more profound changes in the environment such as changing the shape of the enclosure or the room in which the enclosure is placed. Here we are interested a global-remapping that is triggered by entry into a novel environment that the rat has not experienced before. Experimental work has shown that change in the external visual environment causes the grids in the EC to change in a structured manner [32], [33]. Specifically, the grids are coherently rotated and translated, while grid-spacing and field-sizes remain unchanged. The grids generated in the second environment can therefore largely be remapped to the set of grids generated in the first environment via a common rotation and translation, although this match is not exact. The remaining differences between the grids in the two environments could be explained in a number of ways, for example by noise in individual grid rotations, changes in the relative grid origins, or by some combination of the two.

For the purposes of our model we must specify an explicit algorithm for generating changes to the grids as a rat moves through an extended sequence of novel spatial environments. We know that neighbouring MEC cells (such as the group of cells we simulate) have very similar orientations, grid-spacings and field-sizes, but randomised grid origins relative to each other [32]. We make the assumption that moving into a new spatial environment does not disrupt these relationships, so that relative grid orientations, grid-spacing and field-sizes are preserved in each spatial environment. We make the hypothesis that the differences in the grids between two environments that are needed to drive global remapping in our model must therefore arise from changes in relative grid origins. For simplicity we assume that the grid origins are simply re-randomised in each new spatial environment. This is likely to be an over-estimate of how much the grids differ between two environments, at least when considering a single change of the external visual environment while keeping the enclosure unchanged (or changing the enclosure with a fixed visual environment) as has been examined experimentally, and not an extended sequence of completely distinct environments that we consider here. However, this simple algorithm produces changes in EC firing statistics that would be sufficient to drive global remapping in the DG and CA3 in our model while still producing sets of grids with realistic properties in all 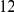 of the spatial environments we simulate.

We model the changes in grid cells due to entry into a novel environment as a two stage process. The grids first undergo a coherent rotation when entering a new environment, subject to some level of noise, where the angle of rotation is randomised. The orientation of the grids relative to some fixed landmark therefore changes but their orientation with respect to each other is largely unchanged. The grid origins are then randomised within this new environment. To generate a set of 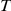 grids, one for each environment, we generate template environment, labeled 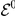, as set out above. A set of environments labeled 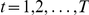 is then generated from this template environment by coherently rotating the grids and then individually randomising each grid origin. The two panels on the right of Figure 2 illustrate this process using the two grids shown in the middle panels.

We assume that our simulated animal lives for one year, which, in rats, is the evolutionarily relevant period for the kind of memory task we investigate, and is actively exploring for around three hours per day. This choice is somewhat arbitrary but not unreasonable and in any case our simulations are largely insensitive to this choice provided that there is enough time for the animal to sufficiently sample each environment before moving on to the next one. We assume that the animal lives for the same length of time regardless of the number of environments experienced, so the length of time spent in each environment is inversely proportional to the total number of environments 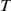. When exploring the animal is assumed to randomly sample the spatial environment, so that we do not explicitly model the pathway that the animal traverses. Again, provided the animal has time to sufficiently sample the spatial environment this choice has little effect on the simulation results.

### Adaptation strategies

As the virtual animal moves around an environment, 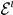, input vectors are generated from the EC using the set of grids associated with that particular environment. The network generates an activity pattern in the DG layer using the winner-takes-all algorithm outlined above. We assume that the network has some target performance level for encoding and decoding new memory patterns which we denote 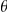. 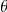 is expressed as a proportion of the total variance in an environment and is therefore dimensionless. The network measures the recent time average of the recoding error and compares it to the target recoding error. If the current recoding error is larger than the target value the network responds by adapting its internal structure in some way. A low recoding error threshold requires the network to encode and decode incoming patterns with high precision. This forces a greater degree of specialisation to each environment by the units in the DG. A high threshold means patterns may be stored and retrieved with less accuracy. This requires less specialisation in the DG. In all cases, when a new DG unit is initialised for any reason its encoding vector 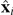 is set to the location in phase space of the current input pattern. A initialised decoding vector is assumed to be identical to the new encoding vector, 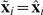. We consider three distinct kinds of adaptation strategy:

- **Conventional synaptic plasticity:** The network starts with a full complement of 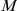 hidden units which are initialised around the origin using an 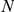-dimensional Gaussian distribution. This produces a network that is not specialised for any particular environment. On entry to the first environment 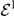 the distribution of encoding and decoding vectors will typically not be well suited to deal with the input distribution and the recoding error will therefore be initially quite high. As the animal explores the environment the network connections are slowly adjusted using a neural gas-like algorithm [37]. In this algorithm the encoding vectors are compared to the input vector and ranked according to how close they lie in input space, and then updated so that they becomes more similar to the input pattern. The magnitude of the updates depend exponentially on the ranking, so that the change in each encoding vector is given by
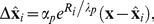
(3)where 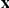 is the input vector, 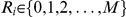 is the rank of DG unit 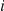, 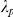 is the decay constant associated with the ranking, and 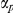 is the overall plasticity scale. We set 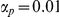 and 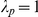, choices which give a reasonable level of plasticity given the statistics of our input. The plasticity described by Eq. 3 causes the distribution of encoding vectors to slowly drift towards the input distribution over time, and the recoding error therefore falls. Unlike Martinez et al. (1993), in our algorithm the rate of learning does not gradually slow. Instead, when the recent-time average of the recoding error (measured over 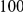 second time intervals) drops below the specified threshold, 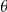, the units become frozen and remain non-plastic until the recoding error rises above 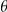, whereupon the connections unfreeze and become plastic again. This typically happens when the input statistics changes due to the network entering a new environment. This process continues until the animal has passed through the full set of 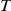 environments.
- **Neuronal turnover:** The network has a full set of 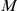 hidden units which are initialised around the origin in the same manner as for the conventional plasticity network. On entry to the first environment the recoding error will be initially high which triggers neuronal turnover, where units are deleted from the network and reinitialised using the current environment's statistics. We set the average rate of neuronal turnover to be 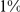 per day and consider two variations of how units are selected for turnover. In *random* turnover units are randomly selected from the network. In *targeted* turnover units all DG units have a internal counter that is incremented each time the unit is activated by an input pattern. The internal counters decay exponentially over time with a half-life of 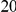 days. When turnover occurs the DG units are ordered by usage, then those with low usage counters are are chosen for deletion before units with higher usage counters. Thus, units that have not been activated by input patterns in the recent past are selected for deletion while units that have been recently activated are preserved. In both cases new units are initialised using the current input vector. The distribution of encoding vectors will therefore gradually change to better represent the new input distribution. We also examine a variation of turnover where reinitialised units are plastic for a period of time following their reinitialisation. In this case, all DG units in the network follow the neural gas rule of Eq. 3 scaled by an additional unit-specific time-dependent scaling factor, 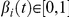. The scaling factor 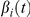 is set to 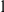 when unit 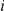 is reinitialised, then decays exponentially over time with a half-life of 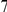 days. When the recent-time average of the recoding error drops below the target threshold turnover ceases until the recoding error rises again, typically this is on entry to the next environment. This process continues until the animal has passed through the full set of 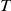 environments.
- **Additive neurogenesis:** On entry to the first environment a single unit is added to the network. As the network has only one DG unit the recoding error is initially very high and this triggers additive neurogenesis, where units are added to the network and initialised using the statistics of the current environment. We set the average growth rate to be 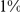 of the maximum DG size per day. Growth continues until the recoding error falls below threshold whereupon neurogenesis ceases. When the animal enters the second environment 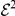, the recoding error will typically rise above the threshold and growth starts once again. This continues until the animal has experienced the full set of 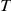 environments. After passing through the 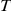 environments the network will have a set of 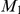 units specialised for environment 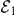, a set of 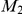 units specialised for environment 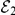, and so on. We impose the constraint 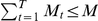 so that the network can never grow larger than the fixed size networks. The neurogenesis network will typically only reach a size comparable to that of the fixed size networks near the end of the animal's lifetime. We also examine a variation where new units are plastic for a period of time following their addition to the network. As with neuronal turnover all DG units in the network follow the neural gas rule of Eq. 3 scaled by an exponentially decaying scaling factor 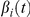, which has a half-life 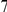 days.

A lower error threshold implies a greater degree of specialisation of the network to each environment. In both the conventional plasticity and neuronal turnover algorithms increased specialisation to each environment is achieved by adjusting the internal connectivity of the network, a process which risks the disruption of retrieval properties when patterns from earlier environments are recalled and decoded. In the additive neurogenesis algorithm the network avoid this problem by adding new connectivity to the network rather than changing existing connectivity. Units that are added in later environments do not affect the retrieval error for earlier environments because they were not used during storage of those patterns. However, a neurogenesis network that becomes over-specialised for the early environments risks using up the entire pool of new hidden layer units too quickly, leaving none remaining for later environments.

## Results

We present three main results in this section. First, we quantify how well the network performs using one of the three different adaptation strategies: conventional plasticity, neuronal turnover (random or targeted), and additive neurogenesis. We also examine pair-wise combinations of these three strategies and finally a combination of all three operating together. Second, we examine the time profile of neurogenesis over the lifetime of the test animal under two different hypothetical rearing conditions: a sequence of twelve environments, representing natural free roaming rearing conditions, and a reduced sequence of four environments, representing laboratory housed rearing conditions. Third, we examine the kind of spatial structure that emerges in DG activity in our model due to the interplay of the grid-cell like EC input patterns and the sparsification that occurs in the DG layer.

For consistency with earlier work, we simulate a network with 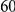 EC cells in the input layer and restrict the DG to have a maximum size of 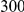 units, a choice that also preserves the experimentally observed 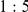 divergence in unit number between the EC and the DG. We choose a target recoding error threshold of 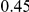. This provides a reasonable target level of recoding accuracy given the network size and the input statistics we use.

### Network performance with different adaptation strategies

In our simulations the network experiences a sequence of twelve environments over a simulated lifetime of one year. The simulated animal spends one month (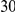 days) in each environment, and is assumed to be actively exploring for around three hours each day. When exploring the animal randomly samples the spatial environment, appearing at a different location every second.

#### Fixed and reinitialising networks

Before we examine network performance using different adaptation strategies we note that there are two kinds of network that are of particular interest to us as reference cases. The first is a fixed size, non-plastic network with a full set of DG units initialised using positive values drawn from an 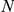-dimensional Gaussian distribution centred at the origin. Such a network will be set up in a completely general way and will not be specialised for any particular environment. The recoding performance of this network provides a measure of how well, in a general sense, a network with 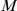 hidden layer units deals with the 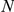 dimensional input governed by the grid cell statistics we have used. We would naturally expect that any adapting network should achieve at least this level of performance, as otherwise the adaptation process is actually lowering the performance of the network and the network would be better off remaining fixed. The second case of interest is a network that is completely reinitialised upon entry to each new environment with a full set of plastic DG units specialised to that environment. Such a network will be completely specialised for the current environment and provides us with a measure of the best possible performance we can expect a network to achieve. As the internal structure of the network changes completely between environments this network also provides us with the worst possible retrieval error we can expect to see.

**Figure 3:**
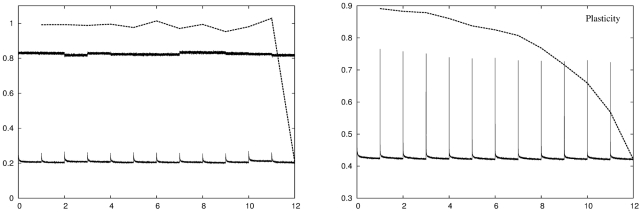
Evolution of the recoding and retrieval errors over 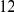 environments for the fixed, reinitialising and plastic networks. Left panel: The recoding error (lower solid line) and retrieval error after adaptation to the final environment (dashed line) of the reinitialising network are a measure of how well a completely specialised network deals with the same kind of statistics. This is the best possible average recoding performance, and correspondingly the worst possible retrieval performance we can expect for a network with 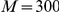 DG units. The recoding error of the fixed network (upper solid line) is a measure of how well a completely generic network deals with the statistics of the spatially driven input we have used. We expect that any adaptation strategy would produce at least this level of recoding accuracy. Right panel: Evolution of the recoding error (solid line) and the retrieval error (dashed line) as a function of environment number for a network that uses a neural gas-like plasticity algorithm with a recoding error threshold of 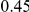. In all subsequent plots we conform to the convention of plotting recoding errors with a solid line and retrieval errors with a dashed line. The errors lie in the range 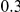 to 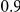 which we also adopt as our standard vertical scale. Conventional plasticity successfully reduces the recoding error in each environment to the target value but only at the expense of increasing the retrieval error for previously stored memory patterns.

The left panel of Figure 3 shows the recoding error for the fixed network and the recoding and retrieval errors for the reinitialising network as the animal moves through a sequence of twelve environments, averaged over 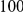 simulations. For the fixed network the recoding error is constant across the entire set of environments. For the reinitialising network the recoding error is initially slightly elevated and then slowly falls as the DG units adjust their distribution to better reflect the new input statistics. We have also plotted the retrieval error for environments 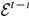 for 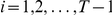, when the network is adapted to the final environment 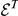. The retrieval error is equal to the recoding error in the final environment, but higher for all preceding environments. A similar retrieval error line may be drawn starting from any of the twelve environments, but the 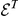 line shows the change in the retrieval error across the whole set of environments. Together, the fixed and reinitialising networks provide a baseline which places the following results in context.

#### Conventional plasticity

We now examine network performance using a conventional plasticity adaptation strategy, where the network is of a fixed size but the encoding vectors of the DG units can change according to a neural-gas like plasticity algorithm. The right panel of Figure 3 shows the recoding and retrieval errors as the animal moves through the twelve environments. When the network enters an environment the recoding error is initially high. Over time, the plasticity of the DG units reorganises the encoding vectors to better represent the new input statistics, reducing the recoding error to below threshold, whereupon plasticity ceases. However, this decrease in recoding error has come at the price of an increase in retrieval error. This increase in error is due to changes in the internal structure of the network which disrupts the representations of earlier environments, and is more pronounced for more temporally distant environments. Conventional plasticity is therefore capable of adapting the network to a change in input statistics but only at the expense of disrupting the retrieval of previously stored memory patterns.

#### Neuronal turnover

**Figure 4:**
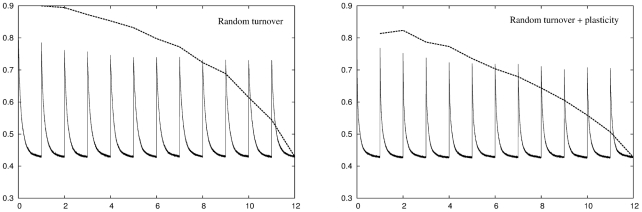
Performance of a network using the random neuronal turnover adaptation strategy across 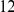 environments. Left panel: Random neuronal turnover successfully reduces the recoding error in each environment to the target level of 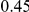 but only at the expense of increasing the retrieval error for previously stored memory patterns. Right panel: Adding conventional plasticity improves network performance but does not qualitatively change this result.

In the neuronal turnover algorithm the network is fixed in size but units in the DG layer may die and be replaced by newly initialised units. There are two variations of this algorithm. In random turnover units are randomly deleted and reinitialised. In targeted turnover units that have not been recently activated by EC input patterns are deleted in preference to those that have been recently activated. The left panel of Figure 4 shows the recoding and retrieval errors across the twelve environments for random turnover with an error threshold of 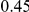. As with conventional plasticity when the network initially enters an environment the recoding error is elevated but, over time, the error is reduced as the network adapts to the new input statistics. Here the adaptation is not due to plasticity of the DG connections but instead is due to units being deleted from the network and replaced by newly initialised units. These newly initialised units tend to have encoding vectors that lie in the more densely occupied regions of input space and therefore better match the input statistics than the units they replace, and the recoding error therefore falls. As with conventional plasticity, random turnover leads to an increase in the retrieval error due to disruption of previously stored representations. The right panel of Figure 4 shows the same information for a network using a combination of random turnover and conventional plasticity. Units that have been turned over in this network are plastic for a few days following re-initialisation. Introducing plasticity increases performance of the network slightly as the encoding vectors are distributed more efficiently, but the results are qualitatively the same.

**Figure 5:**
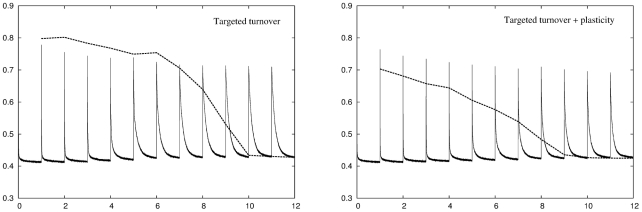
Performance of a network using the targeted neuronal turnover adaptation strategy across 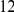 environments. Left panel: Targeted turnover is more successful than random turnover at preserving memory patterns, especially for those stored very recently, but still suffers from a disruption of more temporally distant patterns. Right panel: Adding conventional plasticity improves network performance but does not qualitatively change this result.

Figure 5 shows the same information for a targeted turnover algorithm. A targeted turnover algorithm is more successful than random turnover at preserving previously stored memory patterns, but only the most recently experienced environments can be retrieved and decoded completely accurately. As with random turnover, introducing plasticity improves performance of the network somewhat but does not qualitatively change this result.

#### Additive neurogenesis

**Figure 6:**
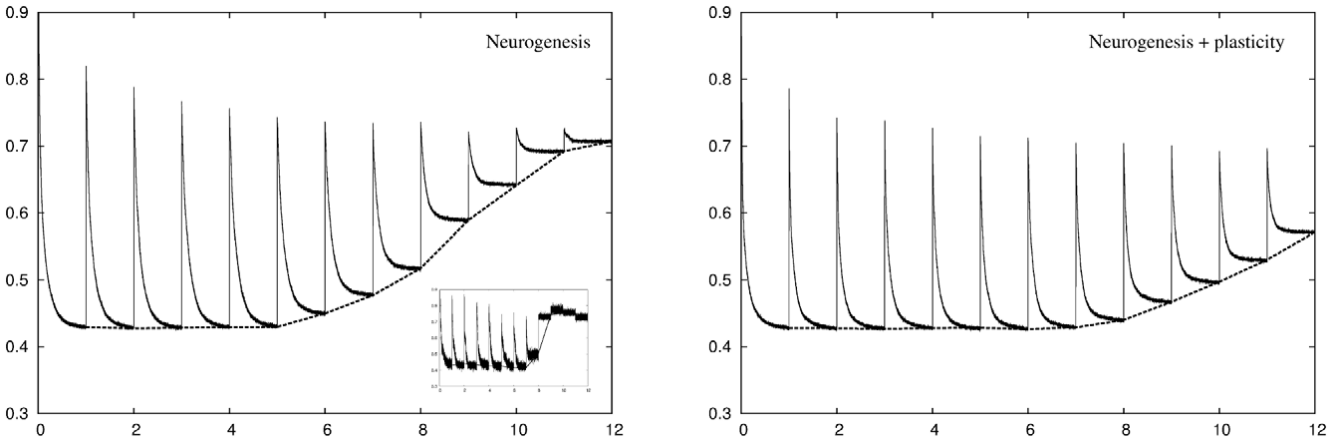
Performance of the neurogenesis network across 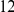 environments. Left panel: For the first five environments the neurogenesis algorithm reduces the recoding error in each environment to the target level of 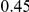 but from the sixth environment onwards the network starts to run out of units to add and the network can no longer achieve this level of performance. The retrieval error for previously stored memory patterns is identical to the recoding error when those patterns were stored, as the internal structure of those parts of the network used to originally encode those patterns does not change over time. Inset: A plot of a single simulation shows how this breakdown of adaptation occurs in a step-like manner when the network runs out of units to add. The gradual degradation in performance shown in the main plot is a result of averaging 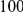 simulations, each of which breaks down at a different point in time. Right panel: Plasticity allows the network to make better use of the units it grows with the result that the network can, on average, deal fairly well with all twelve environments.

We now examine an additive neurogenesis algorithm where the network starts out with a reduced number of DG units but is capable of adding more units over time. The left panel of Figure 6 shows the recoding error across twelve environments. On entry to a new environment we see the characteristic behaviour of an initially elevated recoding error which is reduced over time as the network adapts. Here the adaptation is due to the growth of new units in the DG layer which expands over time. As discussed above, a low recoding error threshold can cause the network to exhaust its capacity for growth by using all its units in the early environments. We see in the inset that this occurs part way through environment six. As there are no more units to add in the final three environments the performance in those environments is very poor. However, in all cases the retrieval error is identical to the recoding error as, although the network is growing, the parts of the network that were used to encode patterns in earlier environments do not change over time so there is no rearrangement of internal structure and therefore no disruption of previously stored patterns. This is the key advantage of using an additive neurogenesis compared to a conventional plasticity or neuronal turnover algorithm. The right panel of Figure 6 shows the same network except that newly grown units are plastic for a few days following initialisation. Plasticity allows the network to use new units more efficiently with the result that the network can deal fairly well with the full set of twelve environments, although after environment seven the network still does not always achieve the target recoding error.

#### A combined adult neurogenesis, turnover and plasticity algorithm

So far we have examined three distinct adaptation strategies, in the form of conventional plasticity, neuronal turnover, and additive neurogenesis, as well as combinations of turnover or additive neurogenesis with plasticity. We have shown that additive neurogenesis allows the network to adapt to new input statistics without disrupting previously stored memory patterns. However, we have also shown that plasticity in the new cells allows them to adjust their synaptic connections and better match the input statistics, thus maximising the benefit of each unit, and that cell death allows units that have previously been added but have since become redundant in the network to die and make space for new units. Each of these three adaptation strategies therefore has its own advantages and disadvantages. Interestingly, experimental results suggest that new granule cells in the DG have a period of enhanced plasticity and that there is a significant amount of cell death within the DG layer as part of its growth. We are therefore motivated to examine a more sophisticated neurogenesis rule that combines all three processes together.

**Figure 7:**
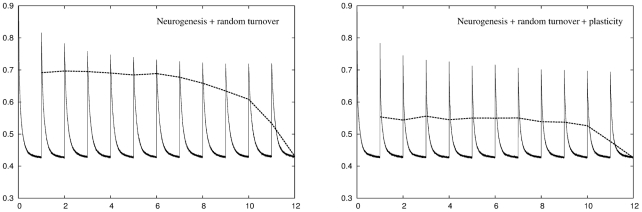
Performance of a network using a combination of neurogenesis and random turnover across 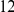 environments. Left panel: The more sophisticated algorithm successfully achieves a recoding accuracy of 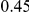 for all twelve environments but once again suffers from an increased retrieval error. Right panel: Adding plasticity improves network performance considerably resulting in a network that can deal with all twelve environments while producing a retrieval error that is consistently lower than either conventional plasticity or neuronal turnover algorithms operating alone.

The left panel of Figure 7 shows the recoding and retrieval errors for a network using a combination of additive neurogenesis and random turnover across twelve environments. In this algorithm the DG grows according to the additive neurogenesis rule presented earlier except that, instead of growth ceasing when the DG reaches the maximum size, units are randomly turned over to make space for new units. The right panel of Figure 7 shows the same network with plasticity. As with the simple additive neurogenesis rule this adaptation is due to the growth of new units in the DG layer. Unlike the purely additive algorithm introducing a degree of neuronal turnover allows the network to achieve the target recoding accuracy in all twelve environments. This once again leads to an increased retrieval error because the network is once again changing its internal state over time, although the magnitude of this error is lower compared to both conventional plasticity and neuronal turnover algorithms operating alone. Adding plasticity to this algorithm allows the network to make much more efficient use of new units, producing a network that is capable of dealing with all twelve environments while at the same time preserving excellent representations of earlier environments. The retrieval error is approximately constant across all previous environments, suggesting that this algorithm might be appropriate if the network were operating as a longer-term memory store.

**Figure 8:**
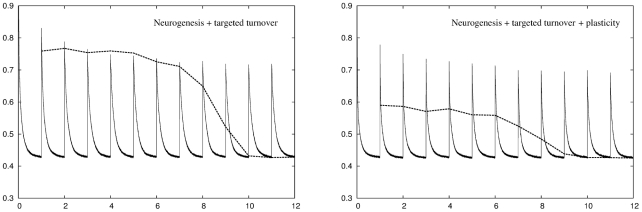
Performance of a network using a combination of neurogenesis and targeted turnover across 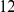 environments. Left panel: The algorithm achieves a recoding accuracy of 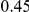 for all twelve environments. The retrieval error is the same as the recoding error for the three most recent environments then increases sharply for temporally more distant environments. Right panel: Adding plasticity improves network performance considerably. The result is a network that can deal with all twelve environments while at the same time having a retrieval error that is lower than either conventional plasticity or neuronal turnover algorithms operating alone.

Figure 8 shows the same information for a network using neurogenesis and targeted turnover. This network is also capable of achieving the target recoding accuracy in all twelve environments. Again, there is an increase in retrieval error due to units dying and being replaced. In this network this is done in a targeted manner, so that units that have not been recently used die off first. The retrieval error is the same as the recoding error for the most recent three environments, but then rises fairly sharply as we examine more temporally distant environments. This algorithm might therefore be appropriate if the network were operating as a short term memory that placed more emphasis on more recent environments compared to most temporally distant ones.

### Time course of neurogenesis

We now turn our attention to the time course of neurogenesis over longer periods in the lifetime of our simulated rat. We are interested in these growth profiles for two reasons. Firstly, it explains the time course of the recoding error for the neurogenesis network we studied in the preceding section in terms of adding units to a growing DG. Secondly, the profiles display several distinct features that together form a specific theoretical prediction of our model which could be used to test our theory experimentally.

**Figure 9:**
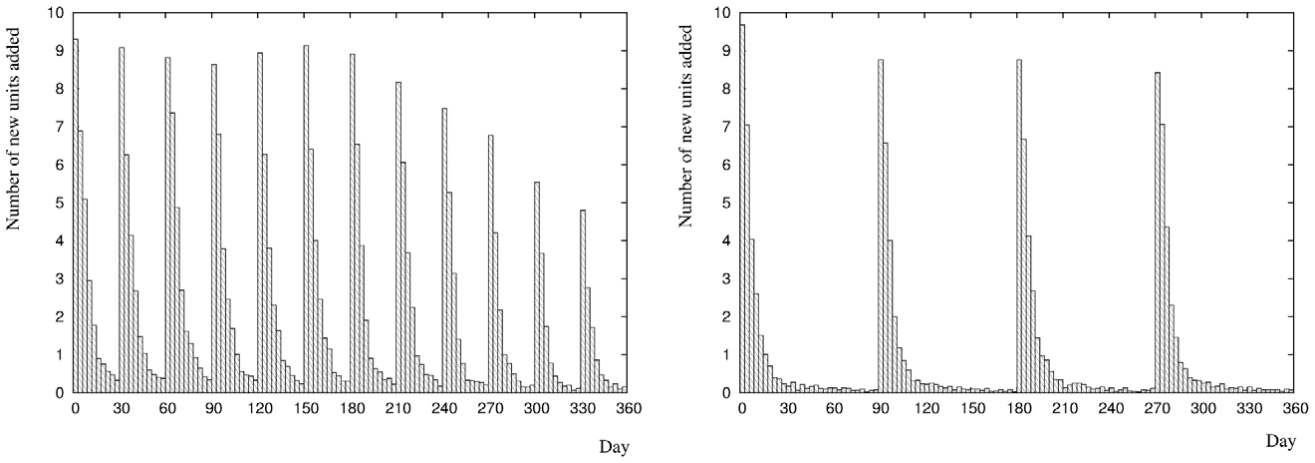
Time course of neurogenesis in the dentate gyrus. Left panel: Neurogenesis profile across twelve environments. On entry to each new environment there is an approximately exponentially decaying growth period. Later environments need fewer new units to achieve the same level of recoding error, indicated by the progressively lower peaks. This reflects an increasing level of generalisation in the network that permits the re-use of existing units. Right panel: Neurogenesis profile across four environments. The same trends of an exponentially decaying growth pattern and a reduction in the number of units added in the later environments compared to the earlier environments can be seen. However, the mean overall level of growth for four environments (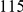 units) is lower compared to the mean overall level of growth for twelve environments (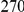 units).

Figure 9 shows the growth profile of the DG for two hypothetical rearing conditions averaged over 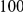 simulations. In the left panel the network experiences a sequence of twelve environments. On entry to each new environment there is an approximately exponentially decaying period of growth. Once enough new units have been added to bring the recoding error below the target error of 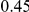 growth slows down dramatically, although it does not stop entirely as fluctuations mean new units do continue to be added. This growth pattern that is repeated on entry to each subsequent environment. The overall amount of growth is smaller in later environments compared to earlier environments. This is because the network begins to build up experience of a larger and larger number of environments and begins to find more and more similarities between the statistics of each new environment and the statistics of the set of environments it has already experienced. This process of generalisation means that fewer units must be added on entry to later environments to achieve the same target level of recoding error.

The right panel of Figure 9 shows the growth profile across a more restricted sequence of four environments. This simulation represents typical, laboratory housed rearing conditions where the animal experiences very few environments over its lifetime. Again, we see an approximately exponential growth pattern within each environment, and that the degree of growth is slightly lower in the last environment compared to the first one. However, the overall level of growth in the dentate gyrus is lower, with an average of 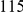 units needed to deal with four environments compared to 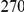 units needed to deal with twelve environments. Note that the number of units required does not scale linearly with the number of environments due to the re-use of existing units in later environments.

The shape of the neurogenesis profiles is strongly dependent on our choice of additive neurogenesis rule. We have limited the average rate of growth to 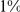 of the maximum DG size per day, a choice that is broadly consistent with the experimental data. As the amount of neurogenesis is typically measured over much longer periods the experimental data does not provide the temporal resolution to constrain the rule with anything more than an average rate of growth. We have also explored the situation where each environment is distinct from the previous environments. There is therefore very little overlap in the statistics of each environment. We do not therefore expect an exact quantitative agreement between our model and experimentally measured neurogenesis profiles. However, we expect that the principle results would be preserved. Our model predicts that growth of the DG should typically be triggered by entry into novel environments that an animal has not experienced before. This growth should follow an approximately exponential time course, and later environments should typically induce less growth compared to earlier environments. We also predict that the final size of the DG for animals raised under conditions where fewer environments are experienced, for example in laboratory housed rearing, will be smaller compared to animals raised under richer, more complex rearing conditions.

### Place cells in DG

Modeling work has shown that a network performing sparsification on spatially structured input can generate place-cell like receptive fields [38]. In the model presented in this paper we have used a phenomenological model of grid cells in the EC to generate input firing patterns that are passed to the DG which performs a sparsification operation. We are interested to see if the sparsification step in the DG of our network also leads to spatially dependent firing in the dentate gyrus, and if this spatial dependence has similarities to the well-documented phenomenon of place-cells [36]. Of particular interest is an evaluation of how spatially selective units in the DG are and how this selectivity changes and develops as the animal moves between different spatial environments.

We examine the spatial firing properties of a group of DG cells in simulations similar to those presented in the results section. Briefly, the network explores two distinct spatial environment for a period of one month (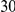 days), adapting its internal structure to cope with the changes in input statistics that occur when it move from environment one to environment two. We record DG activity as the animal moves into the second spatial environment at time points of 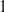 day, 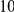 days, 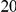 days and 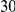 days. We examine a network using the additive neurogenesis with plasticity algorithm, but results for a network that uses additive neurogenesis in combination with some form of neuronal turnover (either targeted or random) are qualitatively very similar.

**Figure 10:**
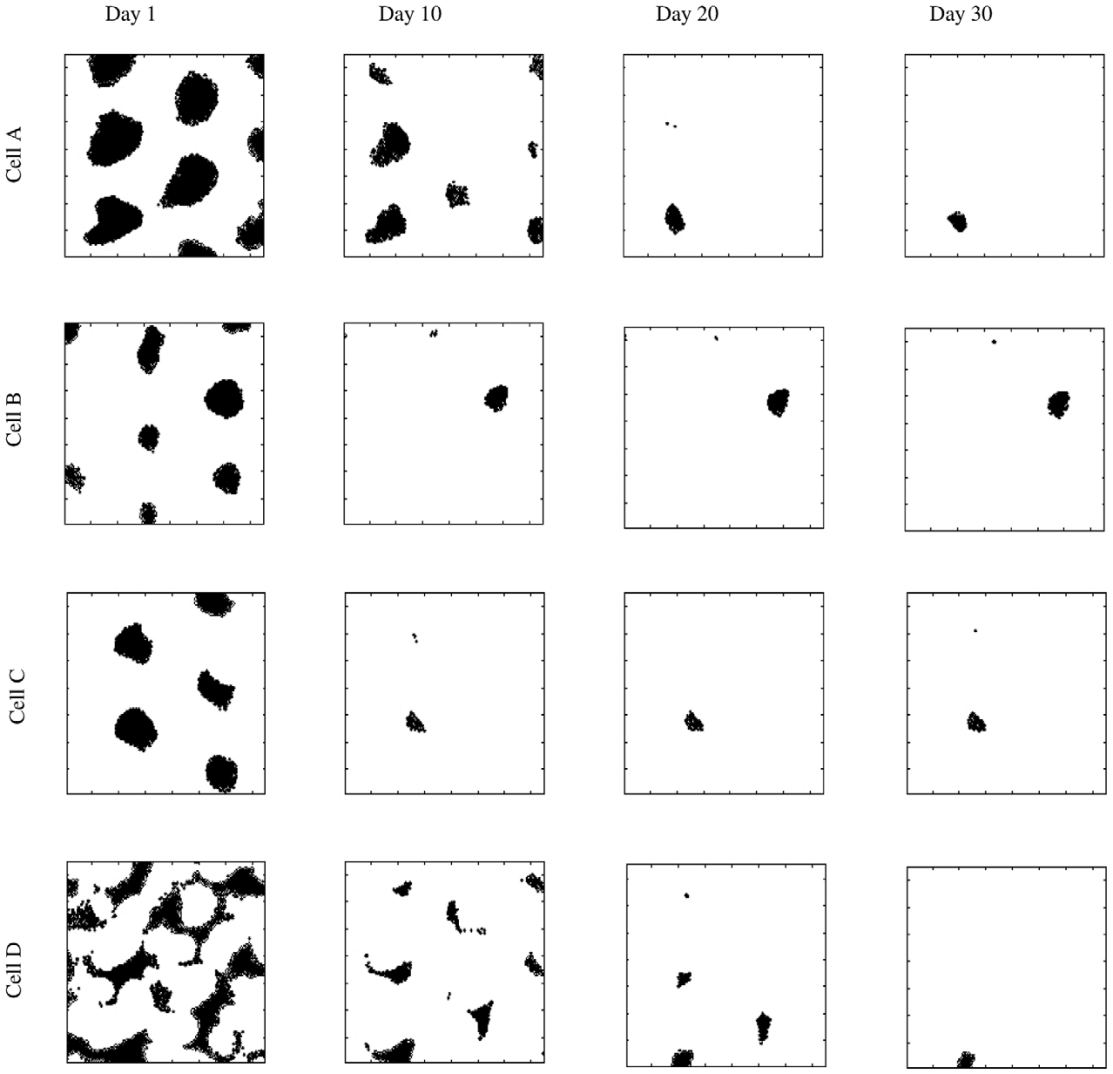
Development of spatial dependence of activity in the dentate gyrus layer of our network upon entry into a novel environment. We show four cells (from top to bottom) at four different time points (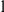, 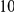, 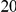 and 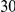 days, from left to right). Left column: After 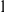 day in the new environment each DG cell us activated by a large area of the spatial environment. Middle left column: After 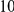 days a degree of refinement has occurred and the place fields have become more restricted. Middle right column: After 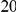 days further refinement leads to activation patterns that resemble place cells in the DG. Right column: After 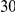 days the final response of the cells are very similar to experimentally observed place cells with one main place field and occasionally some scattered areas of secondary activation. The network uses an additive neurogenesis with plasticity algorithm, but results are qualitatively the same for any of the four variations of neurogenesis we explored in the results section.

Figure 10 shows the spatial firing response of four typical DG units at four time points in the second environment. After one day the input statistics have only recently changed due to entry into the novel second environment and only a few DG cells have so far been added to encode newly active regions of input space. Each unit therefore handles a relatively large area of input space which translates to it being activated by a large proportion of the spatial environment. By day 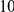 further addition and optimisation of new units has increased the size of the DG layer and the initially broadly tuned response has refined into a number of distinct but still quite large place fields. As more units are added these place fields are refined further, until by day 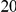 we have responses that resemble place fields as seen in experimental literature. After 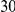 days we have a mature network and the cells are left with one main place field and occasionally some scattered areas of secondary activation.

We have shown the development of a set of four fairly typical DG cells in our network. There is a great deal of variety in the number and size of place fields that the DG cells have, with some having a single place field (as in the examples shown), others more than one, and yet others having no place fields at all. The resulting place fields are fairly realistic in appearance and correspond well with experimental observations. However, we should point out that the time scales involved in the emergence of place fields in our model are rather long compared to those observed in experimental work. Typically, place cells develop and refine over the course of a few minutes rather than days when a rat enters a new environment. There are several possible reasons for this discrepancy in timescales. Perhaps most importantly, we have made several simplifications when constructing our model hippocampus, adult neurogenesis rule, and the statistics of the environments which the rat experiences. Although the behaviour of our model is qualitatively insensitive to many of these choices, these choices have important consequences if we wish to interpret our results in a quantitative way. For example, the statistics of the two environments we have used are rather different from each other. If they were very similar we would expect that less refinement would be required and recognisable place fields would emerge much faster. We have also made assumptions about the amount of neurogenesis per day, limiting it to an average of 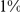 of the maximum DG size per day. If new units were added at a higher in the first few days after entry to an environment compared to later times, then refinement would also be much faster. Addressing these issues thoroughly is beyond the scope of this current work. However, we are still able to show in a qualitative sense that place-cell like responses can can emerge and refine in our model as a natural result of the sparsification operation of the DG operating on spatially structured EC input.

## Discussion

In this paper we have extended our earlier model of additive neurogenesis in the hippocampus to include realistic, spatially driven input firing patterns in the form of grid cells in the entorhinal cortex. We confirmed our earlier finding that additive neurogenesis is a superior adaptation strategy compared to conventional synaptic plasticity or neuronal turnover. We have also shown that a more sophisticated neurogenesis rule that incorporates both cell death and enhanced plasticity of new granule cells is superior to any one of the three individual strategies operating alone.

We focus on the interaction of the EC and DG layers, and leave the remaining layers of the hippocampal network implicit. The DG in our network encodes input patterns and forms a sparse, binary representation of the continuous, non-sparse EC input. We assume that CA3 then carries out a storage and retrieval function, and that CA1 decodes the retrieved pattern, without modeling these processes explicitly. A more complete hippocampal model would include an explicit CA1 decoding layer and an explicit model of storage and retrieval in area CA3, perhaps via a Hopfield network. However, we do not believe that including an explicit model of CA3 and CA1 will change the principal findings presented in this paper. This is because, regardless of what happens downstream of the DG, incoming EC input patterns will still be encoded in the DG and the network will still face the problem of having to adapt the DG encoding to changes in the EC statistics without disrupting previous encodings and the retrieval of existing memory patterns.

The nature of the rule governing the addition of new units to the dentate gyrus has profound consequences for network performance. In the absence of experimental data we have opted for a simple approach where new units are added if the average network performance falls below a given threshold. We also limit the average rate of of neurogenesis to the experimentally observed long-term average of 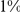 of the maximum DG size per day. The benefit of this simple rule is that we do not require the network to have knowledge in advance of the maximum size that the dentate gyrus can grow to, nor the number or complexity of environments it will experience. The additive neurogenesis rule could in principle be extremely complicated and take into account the current size of the dentate gyrus and the details of the environments the network has already experienced or, as we believe more likely, it could be a relatively simple rule that has evolved into a form which is appropriate for the typical life experiences of the animal in question. Certainly there is a strong evolutionary pressure to optimise this rule as the advantages of a properly functioning memory system are profound. Animals with more efficient adult neurogenesis rules would therefore be at considerable advantage compared to those with less efficient rules. Another possibility is that the neurogenesis rule is dynamic, reacting to low levels of growth by lowering the recoding error threshold or high levels of growth by raising the recoding error threshold. Such a rule seems appropriate as the animal does not know in advance how many and how complex the environments it will experience. An animal that finds itself experiencing a limited number of environments might be better off using more DG units to improve the representation of those environments rather than save units for future environments that it may possibly never experience. This kind of neurogenesis rule would lower the difference in final dentate gyrus size between the laboratory housed and natural rearing strategies. Adult neurogenesis could also occur at a variable rate, perhaps depending on the difference between the measured and target recoding errors. This would make the growth profile steeper in the first few days after entry to the environment, so that the network adapts much more quickly to a new environment. It would also cause place cells in the dentate gyrus to refine much more quickly. Both possibilities are consistent with the experimental data on growth rates which do not have the temporal resolution needed to make a strong statement about the time course of growth. However, we do not believe that the details of the adult neurogenesis rule will change in a qualitative way any of the results presented here. The advantages of the neurogenesis strategy stem from the fundamentally different approach to dealing with the changing statistics of the input patterns. A network that uses conventional plasticity is faced with the problem of having a complete network from the start and having to decide which parts of that network to adapt. We have found in a variety of cases [29], [30] that, while the network can adapt, it is almost certain to disrupt or even completely destroy the retrieval properties of that network. The same is true for a network that uses neuronal turnover for essentially the same reasons. In contrast, with additive neurogenesis the network can patiently add new units and grow in response to changes in the input environment as necessary, a process which does not disrupt retrieval of memories that have already been stored.

Experimentally it is known that the amount of neurogenesis decreases over the lifetime of the animal. The reasons for this decrease are not clear but there are several possible explanations. It may be due to the increasing generalisation of the network over time. As more and more environments are experienced the network adds more units to the DG, each specialised for one of the environments. Over time the distribution of the DG encoding vector begins to reflect the statistics of the ensemble of environments. Later environments are more likely to be adequately handled by the existing set of encoding vectors and fewer new units therefore need to be added. A second possible explanation lies in the statistics of the sampling of the ensemble of environments. If an animal experiences a set of 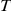 environments over its lifetime, and it randomly sampled these environments at the rate of one per day, then at first the animal would expect to see a different environment almost every day. As time progressed it would become more and more likely to revisit an earlier environment compared to visiting a novel environment. Less neurogenesis would be needed in the later stages of life because the animal would only rarely enter a novel environment. A third possibility is that the level of cell proliferation in the hippocampus simply decreases over the lifetime of the animal. The effect of this would be to place more emphasis on earlier environments compared to later ones as more units would be available in those early environments, but this would be a viable strategy if the earlier environments were generally considered to be more important than later environments. It is likely a combination of all three that leads to the reported decrease in neurogenesis. In our simulations the statistics of each environment is generated based on experimental data on grid cell remapping in response to a single change in the visual environment. It it not known exactly how the grid cells change when a rat moves through an extended sequence of environments. In the absence of this data we have chosen to generate each of our environments independently, so that each environment tends to be very different from every other environment. The degree of generalisation we see in our simulations is therefore fairly low. However, our simulation results are still qualitatively consistent with the experimentally observed trend. One of the advantages of our model is that we can push our simulations to the extreme in a manner that is not possible experimentally and show to what extent the DG can generalise on the kind of statistics generated by grid cells. We will explore this issue further, and investigate the relative contributions of other mechanisms to the decrease of neurogenesis over the lifetime of an animal, in future work.

Although a large number of new granule cells are produced in the rat dentate gyrus each day, only a small number survive and are integrated into the hippocampal network. Cells that do not become integrated die within a few days. A large amount of cell death therefore occurs in the dentate gyrus and is a natural part of the neurogenesis process. In our model neurons are added to the DG when the current recoding error rises above the target level at an average rate of 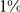 of the maximum DG size per day. Experimentally it has been shown that it takes at least one to two weeks for a newly born cell to mature to the point at which it becomes recruitable by the network [16], [39], [40]. This time scale is too long for units to be produced only when needed. There must therefore be a pool of new neurons available at all times, of approximately the right size, to ensure that the dentate gyrus has granule cells available to be recruited when needed. During periods where no units are recruited neurons in the pool will simply die off and be replaced the next day. Periods with very low recruitment levels typically happen in the second half of each epoch spent in an environment, after the network has already adapted to that environments input statistics. In the simple additive neurogenesis algorithm this cell death occurs only amongst new granule cells that are not recruited into the network. In the more sophisticated algorithms that combine neurogenesis and neuronal turnover there is an initial period where cell death is restricted to new unrecruited granule cells. After the DG reaches its maximum size older cells begin to die off if they have not been activated recently in order to make room for new cells. There is therefore a distinct transition from a regime where only new, unrecruited granule cells die to a regime where both new cells and existing granule cells die. The greater the rate of neurogenesis the sooner in an animals life the DG will reach its maximum size and the sooner this transition will occur. Experimentally, it is still unclear to what extent existing granule cells contribute to cell death in the DG. It is an interesting prediction of our model that both should occur and that there should be a transition between two regimes: in the early periods of an animals life cell death should be accounted for mainly by new granule cells, while in later periods of an animals life it should be accounted for by a mixture of new and existing granule cells.

Our model is based on data from the rodent hippocampus. In particular we used a model of grid-cell firing in the EC of rats to generate input patterns that were encoded by the DG. There is evidence for the presence of grid-cells in human hippocampus [41], and place cells have been reported in both primate and human hippocampus [42]–[44]. Spatially dependent activity therefore seems to a part of hippocampal input and of firing downstream in the DG and CA3 in both of these species. It is therefore natural to ask to what extent can we relate our findings on neurogenesis to other species. This is not a trivial question to answer, as there are differences in adult neurogenesis between rats and even other rodents such as mice. In rats, for example, there is strong evidence that the dentate gryus grows, at least during the first few months of life [5], [45], [46] which does not seem to be the case in mice [47], although in terms of cell numbers the possible quantitative contribution of adult neurogenesis is considerably lower than the variance between animals of the same age, so that a small net growth cannot be excluded. The long-term persistence of adult-born hippocampal neurons certainly argues in favor of their lasting functional contribution [2], and there is also data based on a genetic lineage-tracing model that supports the idea of net growth in the mouse [3]. Differences in adult hippocampal neurogenesis between other species are even more remarkable. Several bat species show little to no adult hippocampal neurogenesis [48] whereas foxes have a particularly large number of immature cells despite having few new neurons [49]. However, it is difficult to draw general conclusions as very few species have been studied in detail. Data on adult hippocampal neurogenesis in humans are particularly scant [50], although we have hypothesised that, based on a large post-mortem study, humans and mice might actually be quite similar with respect to adult hippocampal neurogenesis [51]. Explanations of these differences have been put forward in the literature from an evolutionary perspective, citing influences such as the demands of spatial navigation [52] or the pressure for orientation and exploration [53]. At present it is difficult to make firm conclusions about relative and absolute levels of neurogenesis in different species. However, the model we have presented based on the rodent literature provides an excellent starting point for a comparative analysis as more data become available.

In conclusion, we have taken our earlier model of a hippocampal memory circuit and examined network behaviour across a series of environments using realistic spatial input in the form of grid cells. We have shown that allowing DG units to be plastic, or allowing neuronal turnover where units in the network to die and be replaced by new ones, were inappropriate ways of adapting the network to changes in the input statistics between environments, as both methods disrupted the retrieval and correct decoding of previously stored patterns. An additive neurogenesis strategy, where the network starts out with fewer initial units but grows over time, allows the network to accommodate changes in the input statistics while preserving representations of earlier environments. However, an additive neurogenesis rule could not always deal with the the full set of environments as it did not know in advance the number and complexity of environments it would experience and would sometimes exhaust its potential for growth too early in the animals lifetime. A more sophisticated neurogenesis rule that incorporates both cell death and enhanced plasticity of new immature granule cells solved this problem and is superior to any one of the three individual strategies operating alone. If cell death occurs randomly then the network has a similar level of performance when decoding memories from any earlier environment, as might be appropriate if the hippocampus were operating as a long-term memory store. If cell death is targeted, so that unused DG units tend to die off and more active units are preserved, then more recently encoded patterns are decoded more accurately than more temporally distant one, as might be appropriate if the hippocampus were operating as a short-term memory store. We have also generated growth profiles for the DG over the lifetime of an animal under different hypothetical rearing conditions. These profiles generate several distinct theoretical predictions that can be used to test our theory experimentally. Finally, we have explored the formation, development, and stability of place cells in the dentate gyrus, and shown they could emerge and refine in a natural manner. Sparsification in our model is interpreted as a preprocessing step performed on behalf of CA3 [38] which raises the possibility that place cells in the DG might be a result of sparsification operating on spatial input, rather than a specific computational goal of the network.
